# The Efficacy and Safety of Rituximab in Patients with Idiopathic Inflammatory Myopathy-Associated Interstitial Lung Disease: Case Series

**DOI:** 10.3390/jcm12103406

**Published:** 2023-05-11

**Authors:** Youngeun Jang, Hee-Young Yoon, Hyun-Sook Kim

**Affiliations:** 1 Division of Allergy and Respiratory Diseases, Department of Internal Medicine, Soonchunhyang University Seoul Hospital, Seoul 04401, Republic of Korea; jyeun5439@naver.com; 2 Division of Rheumatology, Department of Internal Medicine, Soonchunhyang University Seoul Hospital, Seoul 04401, Republic of Korea

**Keywords:** rituximab, myositis, lung diseases, interstitial, respiratory function tests, steroids

## Abstract

Idiopathic inflammatory myopathy (IIM)-associated interstitial lung disease (ILD) is often rapidly progressive with a poor prognosis; however, no standard therapeutic regimen has been identified. This study aimed to investigate the efficacy and safety of rituximab in IIM-ILD patients. Five patients who had been administered rituximab for IIM-ILD at least once between August 2016 and November 2021 were included. Lung function decline was compared one year before and after rituximab. Disease progression, defined as a greater than 10% relative decline in forced vital capacity (FVC) compared to the baseline, was also compared before and after treatment. Adverse events were recorded for safety analysis. Five IIM-ILD patients received eight cycles. FVC-predicted values significantly decreased from 6 months before rituximab administration to those at the baseline (54.1% predicted (pre-6 months) vs. 48.5% predicted (baseline), *p* = 0.043); however, the FVC decline stabilized after rituximab. The rate of disease progression before rituximab showed a tendency to decrease after rituximab (75% (before) vs. 12.5% (6 months after, *p* = 0.059) vs. 14.3% (12 months after, *p* = 0.102)). Three adverse events developed, but none resulted in death. Rituximab can stabilize lung function decline with tolerable safety in Korean IIM patients with refractory ILD.

## 1. Introduction

Idiopathic inflammatory myopathies (IIM) are a heterogeneous group of rare, acquired disorders characterized by inflammation of skeletal muscle, presenting muscle weakness, elevation of muscle enzymes, and biopsy-proven muscle inflammation [1,2,3]. The classification of IIM classically includes dermatomyositis (DM), polymyositis (PM), and inclusion body myositis [3,4], as well as myositis of the antisynthetase syndrome (ASS) and immune-mediated necrotizing myopathy [5,6]. Interstitial lung disease (ILD) is a common pulmonary manifestation of IIM, observed in 20–40% of cases [7,8,9]. The prognosis of IIM-ILD varies depending on the type of IIM, with rapidly progressive ILD being associated with a poor prognosis, particularly in older patients, those with anti-melanoma differentiation-associated gene 5 (anti-MDA 5) antibodies, cardiac involvement, decreased blood lymphocytes, a low ratio of the partial pressure of arterial oxygen to the fraction of inspired oxygen, and hypoalbuminemia [10,11,12,13,14]. Although there is no consensus on the specific treatment regimen for IIM-ILD, glucocorticoids or glucocorticoids with immunosuppressants are considered cornerstones of treatment for IIM-ILD [15,16]. Glucocorticoids have traditionally been the first-line treatment, but due to the insufficient response to glucocorticoid monotherapy [17], the combination of glucocorticoids with immunosuppressants is often preferred for steroid-sparing effect or improved efficacy in patients with rapidly progressive or refractory ILD [15,16].

Rituximab is a chimeric monoclonal antibody targeting the CD20 protein on B-cells, leading to B-cell depletion [18]. In autoimmune diseases, B-cells play an important role in disease pathogenesis, such as the secretion of autoantibodies or inflammatory cytokines, autoantigen presentation, and interaction with T cells [19]. In various immune-mediated conditions, including rheumatoid arthritis and anti-neutrophil cytoplasmic antibody-associated vasculitis [20,21], the efficacy of rituximab has been demonstrated in controlling disease status. Moreover, real-world data for the beneficial role of rituximab treatment in IIM-ILD have been recently reported, showing the stabilization of lung function and regression of lung lesions on high-resolution computed tomography (HRCT) [22,23,24,25,26,27,28,29,30]. However, most studies have included few patients, and relatively scarce data are available for Asian individuals. Herein, we investigated the efficacy and safety of multiple cycles of rituximab in Korean patients with advanced IIM-ILD.

## 2. Materials and Methods

### 2.1. Subjects

Five patients diagnosed with IIM-ILD at Soonchunhyang University Seoul Hospital, Seoul received at least one cycle of rituximab between August 2016 and November 2021. IIM was diagnosed by a rheumatologist according to the revised classification criteria of the European League Against Rheumatism/American College of Rheumatology [31]. We defined refractory or uncontrolled IIM as cases showing persistent decline in pulmonary function, worsening clinical symptoms, and increased lesions on HRCT even when treated with first-line therapy, such as steroids, in clinical practice. ILD was diagnosed based on HRCT and/or pathological findings. The study was approved by the Institutional Review Board of the Soonchunhyang University Seoul Hospital (2022-08-020), and the requirement for written informed consent was waived due to the retrospective study design.

### 2.2. Clinical Data

Clinical data at the time of rituximab administration were retrospectively obtained from medical records. Spirometry and diffusing capacity for carbon monoxide (DLco) were measured according to the American Thoracic Society (ATS)/European Respiratory Society recommendations [32,33,34]. Comorbidities were assessed via a chart review.

Cardiac catheterization is used to confirm the presence of pulmonary hypertension when the mean pulmonary arterial pressure is equal to or exceeds 25 mmHg at rest. We also investigated the complications and prognosis after rituximab administration through a retrospective chart review. Rituximab-related complications were defined as those occurring within six months of administration. Acute exacerbation (AE) of ILD was defined as an acute, clinically significant respiratory deterioration that characterized new widespread ground glass opacities without cardiac failure or fluid overload [35]. Elevation of serum erythrocyte sedimentation rate (ESR, >20 mm/h) and C-reactive protein level (CRP, >0.5 mg/dL) was collected to identify inflammation.

Pulmonary function tests (PFTs) were conducted usually every three months during the follow-up, and data on PFTs were collected one year before and after rituximab administration. HRCT scans were obtained under full inspiration without contrast enhancement. The HRCT images were classified as common ILD-type features, such as nonspecific interstitial pneumonia (NSIP), organizing pneumonia (OP), and mixed NSIP/OP pattern, according to the ATS/ERS classification of idiopathic interstitial pneumonia [36]. Progression was identified on the HRCT as an increase in disease extent of at least 25% between consecutive visits.

### 2.3. Statistical Analysis

All values were presented as medians with interquartile ranges for continuous variables or as numbers with percentages for categorical variables. We evaluated changes in lung function using either continuous or categorical variables. The Wilcoxon signed-rank test was used for continuous comparison of PFTs between each interval. For sensitivity analysis, we analyzed changes in lung function of five patients based on the first rituximab administration dose. To calculate the best fit for the decline in lung function over time before and after rituximab administration, generalized estimating equations (GEE) were used. For categorical comparisons, disease progression was defined using relative decline due to the possibility of underestimation when evaluating disease progression with absolute decline in patients with advanced lung function impairment (forced vital capacity (FVC) < 50% predicted or DLco < 35% predicted) [37]. Disease progression was defined as a ≥10% relative decline in FVC over 6 or 12 months (FVC_baseline_ − FVC_after 6 or 12 months_/FVC_baseline_) or as a ≥15% relative decline in DLco over 6 or 12 months (DLco_baseline_ − DLco_after 6 or 12 months_/DLco_baseline_) [37]. All statistical analyses were performed using the statistical software package SPSS (version 24.0; IBM Corp Armonk, NY, USA), and figures were created using GraphPad Prism (version 9.00 for Windows; GraphPad Software, San Diego, CA, USA). *p* < 0.05 was considered statistically significant (two-tailed).

## 3. Results

### 3.1. Baseline Demographics

The five patients had a median age of 40 years, 80% were women, and all were never-smokers (Table 1). Of these, two (40%) and three (60%) had PM and DM, respectively. Three patients were diagnosed with pulmonary hypertension on cardiac catheterization and one patient was supplied with home oxygen. Two patients had a history of malignancy, which were rectal cancer and thyroid cancer, respectively. The most commonly observed antibody was antinuclear antibody (80%), followed by Anti-SSA/Ro (60%), Anti-Jo-1 (60%), and Anti-Ro-52 (60%). The HRCT pattern was NSIP in three patients and OP and NSIP/OP in one each. The median administrated dose of rituximab was 1800 (1225–1975) mg. Three patients received one cycle of rituximab, and the other two patients received two or three cycles of rituximab, respectively. The interval between rituximab administration of patients with three doses was 10 months and 15 months, and that of patients with two doses was 24 months. The median time from diagnosis to rituximab administration was 34.0 (24.8–49.0) months, and the median time from rituximab administration to the last follow-up was 20 (10.3–23.4) months.

Since eight cycles of rituximab were administered, we collected clinical data from the eight rituximab administrations (Table 2). More details are provided in Supplementary Table S1. At the eight cycles, all cases were receiving steroids at a median dose of 7.5 mg. From the eight times of administration of rituximab, four cases received cyclophosphamide, each case received mycophenolate mofetil and methotrexate, respectively. The median FVC and DLco were 48.5% predicted (37.1–56.6% predicted) and 34.0% predicted (24.0–50.0% predicted), respectively. The serum ESR was elevated in all eight (100.0%) cases, whereas the CRP level was not.

Of the eight cases, four (50.0%) cases received rituximab for uncontrolled IIM, and the remaining four (50.0%) received rituximab for ILD progression, including the occurrence of AE (12.5%), progression on HRCT (12.5%), sustained hypoxemia (12.5%), and deterioration of lung function (12.5%).

### 3.2. Comparison of Clinical Parameters between Pre- and Post-Rituximab Administration

**Figure 1:**
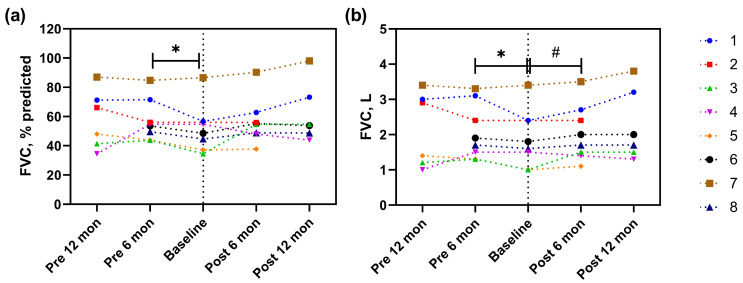
FVC measurements obtained at each visit before and after rituximab in patients with IIM-ILD. (**a**) Changes in FVC% predicted, (**b**) changes in FVC, L. Baseline data were obtained immediately before the initiation of rituximab therapy. Each dot represents the lung function at each visit. Significant at the level * *p* < 0.05, # *p* < 0.1. Abbreviations: IIM, idiopathic inflammatory myopathies; ILD, interstitial lung disease; FVC, forced vital capacity; Pre, pre-treatment; Post, post-treatment.

**Figure 2:**
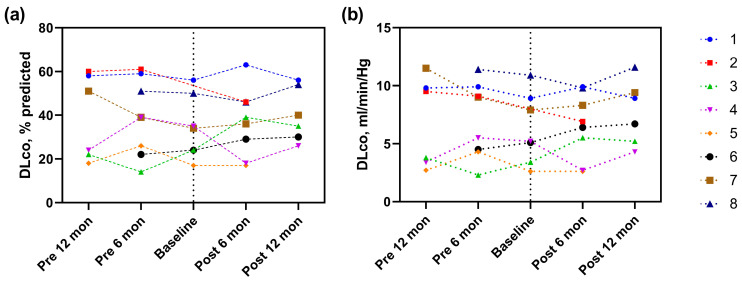
DLco measurements were obtained at each visit before and after rituximab in patients with IIM-ILD. (**a**) Changes in predicted DLco%; (**b**) changes in DLco, min/mL/Hg. Baseline data were obtained immediately before the initiation of rituximab therapy. Each dot represents the lung function at each visit. Abbreviations: IIM, idiopathic inflammatory myopathies; ILD, interstitial lung disease; DLco, diffusing capacity of the lung for carbon monoxide; Pre, pre-treatment; Post, post-treatment.

The FVC% predicted six months before rituximab was significantly higher compared to that at the baseline FVC% predicted (54.1% predicted (pre-6 months) vs. 48.5% predicted [baseline], *p* = 0.043); however, changes in FVC were insignificant from baseline to 6 (54.9% predicted, *p* = 0.128) and 12 months (54.1% predicted, *p* = 0.116) after rituximab administration (Figure 1A and Table 3). Measured FVC also reduced significantly from 6 months before rituximab administration to baseline (1.8 L (pre-6 months) vs. 1.6 L (baseline), *p* = 0.046), but marginally increased from baseline to 6 months after rituximab administration (1.6 L (baseline) vs. 1.9 L (post-6 months), *p* = 0.091) (Figure 1B and Table 3). Both DLco% predicted and measured values decreased numerically before rituximab administration and then increased after rituximab administration, with no statistically significant differences between all intervals (Figure 2A,B and Table 3).

In sensitivity analysis based on five first cycles, predicted FVC increased from baseline (48.5% predicted) to 6 (55.1% predicted, *p* = 0.043) and 12 months (54.6% predicted, *p* = 0.043) after rituximab (Supplementary Table S2). Similar trends were detected in changes in measured FVC (1.8 L (baseline) vs. 2.0 L (post-6 months), *p* = 0.043 vs. 2.0 L (post-12 months), *p* = 0.043). There was no statistical significance in DLco changes before and after treatment, but both predicted and measured DLco values tended to increase compared to baseline after 12 months of treatment (Supplementary Table S2).

**Figure 3:**
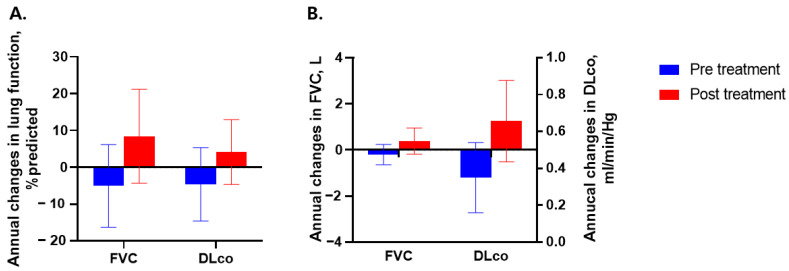
Mean annual changes in FVC and DLco before and after rituximab in patients with IIM-ILD. (**A**) Changes in FVC% predicted and DLco% predicted. (**B**) Changes in FVC, L, and DLco, min/mL/Hg. Data are presented as means and 95% confidence intervals. Generalized estimating equation models were used to estimate annual changes in lung function. Abbreviations: IIM, idiopathic inflammatory myopathy; ILD, interstitial lung disease; FVC, forced vital capacity; DLco, diffusing capacity of the lung for carbon monoxide.

To assess linear changes in lung function, we estimated the annual decline rate in FVC and DLco using GEE (Figure 3). The annual change in FVC% predicted tended to be negative before rituximab administration (−5.0% predicted/year, 95% confidence interval [CI]: −16.2 to 6.2% predicted/year, *p* = 0.380) but changed to numerically positive (8.5% predicted/year, 95% CI: −4.3 to 21.2% predicted/year, *p* = 0.193) after rituximab administration (Figure 3A). Similar trends were observed in annual changes in measured FVC (before: −0.2 L/year, 95% CI: −0.6 to 0.2 L/year, *p* = 0.366, after: 0.4 L/year, 95% CI: −0.2 to 0.9 L/year, *p* = 0.187) (Figure 3B). Annual changes in DLco also decreased before rituximab administration and increased after treatment, but statistical significance was not observed (Figure 3A,B).

**Figure 4:**
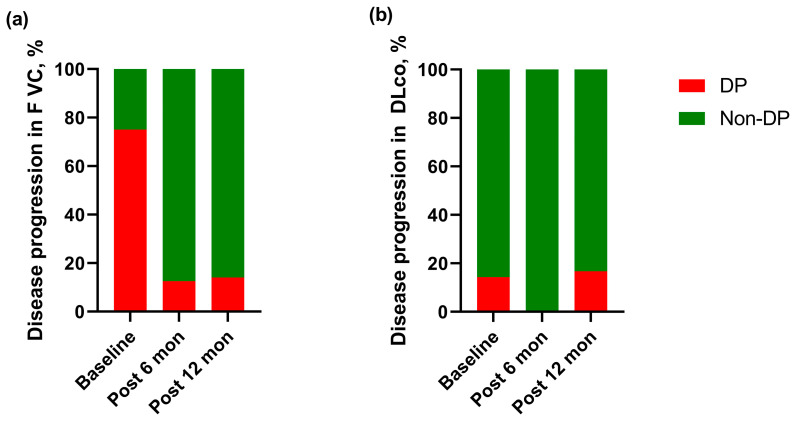
Disease progression in lung function before and after rituximab in patients with IIM-ILD. (**a**) Disease progression for FVC, (**b**) disease progression for DLco. Disease progression was defined as a ≥ 10% relative decline in FVC or a ≥ 15% relative decline in DLco compared with baseline values. Abbreviations: IIM, idiopathic inflammatory myopathies; ILD, interstitial lung disease; FVC, forced vital capacity; DLco, diffusing capacity of the lung for carbon monoxide; Post, post-treatment; DP, disease progression.

The rate of disease progression for FVC during one-year preceding rituximab administration was 75% (6/8) (Figure 4A). However, the rate of disease progression tended to decrease after 6 (12.5%, 1/8, *p* = 0.059) and 12 months (14.3%, 1/7, *p* = 0.102) of rituximab administration compared to that at one year preceding rituximab administration (Figure 4). In HRCT findings, progression was observed in 87.5% (7/8) of cases at the baseline but in 40% (2/5) after treatment (*p* = 0.317). No significant difference was noted in the steroid dose before and after rituximab treatment (prednisone equivalent dose: 7.5 mg/day (before rituximab) vs. 6.3 mg/day (after rituximab), *p* = 1.000).

### 3.3. Adverse Events and Prognosis of Rituximab

Three adverse events related to rituximab occurred, two of which were infection, and one was ileus. Infections included influenza A and COVID-19 infections, which were resolved after proper treatment with antiviral agents. The ileus also improved after supportive care.

Among the eight patients treated with rituximab, none of them died during the entire follow-up period. One patient experienced ILD-AE nine months after rituximab administration and received the second cycle of rituximab 11 months after the first cycle, leading to a stable disease status in ILD.

## 4. Discussion

This retrospective study identified serial changes in the clinical parameters of Korean patients with IIM and advanced ILD who received multiple cycles of rituximab. Rituximab was administered for more than one cycle in five IIM-ILD patients, resulting in a total of eight cases. We observed stabilization of lung function after rituximab administration. After rituximab administration, adverse events occurred in about one third of the cases, but no fatalities occurred.

Ge et al. conducted a study which showed that rituximab improved ILD on HRCT in Chinese patients with anti-MDA5 DM [38]. Among eleven patients, two patients (18%) with mild ILD showed complete remission, and six (55%) patients showed improvement according to a review by radiologists. Although our study did not find a significant improvement in HRCT findings after rituximab administration, we observed a significant reduction in lung function decline among Asian IIM-ILD patients. Given the scarcity of research on rituximab efficacy in IIM-ILD among Asian populations, these findings may support the use of rituximab in Asian patients with refractory IIM-ILD.

We demonstrated that rituximab stabilized the FVC decline in IIM patients with advanced lung function impairment; however, significant increases in FVC were not observed. In previous studies, rituximab therapy was effective for refractory IIM associated with lung involvement [22,23,24,25,26,27,28,29,30]. Andersson et al. reported that rituximab increased FVC from 58% to 72% (*p* = 0.018) and DLco from 17% to 41% (*p* = 0.025) during a median follow-up of 52 months in 24 ASS patients with severe ILD [22]. Additionally, Keir et al. demonstrated that rituximab improved decline in FVC (−13.3% predicted (pre-6–12 months) vs. 8.9% predicted (post-6–12 months), *p* < 0.01) in 33 patients with progressive CTD-ILD, including IIM (*n* = 10) [29]. In our study, FVC did not significantly increase after rituximab administration. The lack of statistical significance in our study may be attributed to several factors. First, the small sample size of participants (8 (our) vs. 24 (Andersson et al.) vs. 33 (Keir et al.)) may not have been sufficient to detect significant differences [22,29]. Moreover, our study population differed from those of previous research in terms of race, with a predominance of Asian individuals, as well as a higher proportion of females (80% vs. 71% vs. 66%) and younger patients (40 years vs. 56.5 years vs. 52.5 years). These differences could potentially affect the effect of rituximab on changes in lung function. Doyle et al. also reported numerically increased changes in FVC (Δ 3.7% predicted) after one year of rituximab administration in 19 ASS-ILD patients without statistical significance (*p* = 0.18) [30], which were in line with our findings.

However, in the present study, disease progression in FVC decreased with marginal significance after rituximab treatment, while disease progression in DLco did not change after rituximab treatment. Decreased DLco is associated not only with parenchymal lung disease, but also with pulmonary hypertension [39]. In pulmonary hypertension, DLco can be decreased due to decreased pulmonary capillary blood volume and pulmonary membrane diffusion capacity due to low cardiac output and increased pulmonary vascular resistance [40]. Compared to previous studies [22,30], our research demonstrated a considerably higher incidence of pulmonary hypertension, with rates of up to 60%. This result may be explained by the inclusion of patients with refractory IIM-ILD, indicating the moderate severity of our study cohort (FVC: 48.5% predicted (our) vs. 58% (Andersson et al.) vs. 57% (Doyle et al.)). Considering these factors, DLco might reflect both severity of ILD and pulmonary hypertension in our study. In ASS-ILD patients with available PFT, FVC was significantly increased (Δ 21.1% predicted, *n* = 7, *p* = 0.016) after 3 years of rituximab administration, while DLco was not (Δ 14.8% predicted, *n* = 7, *p* = 0.25) [30]. Therefore, the fact that the disease progression in DLco did not change significantly after rituximab treatment in our study might be associated with the patients’ medical conditions, particularly pulmonary hypertension, other than the pure severity of ILD.

According to our study, adverse events, including infection, occurred in almost one third of the cases, but all recovered with conservative management. Growing evidence suggests that rituximab can increase the incidence of infection, as rituximab binds to the CD-20 antigen on B-cells, reduces the number and function of B-cells, and affects T-cell immunity [41]. In a previous study, 9 of 25 ASS-ILD patients experienced infections such as pneumonia, influenza, bronchitis, and varicella zoster after rituximab administration [29]. Additionally, in a Norwegian study, 7 of 24 ASS patients with ILD died during a median follow-up of 52 months, and 6 of 7 patients died of infections [22]. In a systematic review of rituximab in the anti-MDA5 dermatomyositis-ILD, infection developed in 37.1% (13/35) of patients after rituximab administration, and of the eleven deaths, four were due to pulmonary infections [42]. Since rituximab is not the first-line therapy in IIM, patients with advanced disease status or refractory disease to first-line therapy are at risk of opportunistic infections because they are typically already receiving several immunosuppressants at the time of rituximab administration [43]. No deaths or serious infections occurred in our study; however, when administering rituximab, consideration of the infection risk and careful post-administration observation may be helpful.

This study had some limitations. First, this was a retrospective observational study in a single center, including a small number of patients due to the rarity of IIM-ILD. The lack of statistical significance in the clinical outcomes may be attributed to the small sample size, and further studies including a larger number of patients may be helpful to overcome this limitation. Second, the indications for rituximab in IIM-ILD have not been standardized, the study patients were heterogeneous and received rituximab for various reasons. However, all patients exhibited ILD progression in terms of lung function, HRCT, or clinical symptoms. Third, the follow-up period was relatively short; therefore, our results may be insufficient to identify the long-term effectiveness or toxicity of rituximab. Long-term follow-up with serial PFT and HRCT may be required to address this issue. Lastly, the analysis of each case of patients who received repeated rituximab due to the small size may result in bias. However, a similar trend was shown in the sensitivity analysis including five patients based on the first rituximab administration. Despite these limitations, our study showed stabilization of lung function with tolerable toxicity after repeated rituximab administration in patients with advanced and progressive ILD and supported the need for further research in larger patient population.

## 5. Conclusions

Rituximab may stabilize lung function decline with tolerable safety in Korean IIM patients with refractory ILD. Further large-scale long-term studies are needed to identify the optimal timing, dose, and response to rituximab.

## Figures and Tables

**Table 1:** Baseline characteristics of the IIM-ILD patients.

Characteristics	Total
Patient no.	5
Age, years	40 (38.0–59.0)
Ever-smoker	0 (0%)
BMI, kg/m^2^	23.5 (18.5–23.9)
Subtype of IIM	
PM	2 (40%)
DM	3 (60%)
Comorbidities	
Pulmonary hypertension	3 (60%)
Diabetes malleus	2 (40%)
Malignancy	2 (40%)
Hypertension	1 (20%)
Antibody	
Antinuclear	4 (80%)
Anti-SSA/Ro	3 (60%)
Anti-La/SSB	1 (20%)
Anti-Jo-1	3 (60%)
Anti-Ro52	3 (60%)
HRCT pattern	
NSIP	3 (60%)
OP	1 (20%)
NISP/OP	1 (20%)
Home oxygen use	1 (20%)
Rituximab dose, mg	1800 (1225.0–1975.0)
Rituximab cycle	
1	3 (60%)
2	1 (20%)
3	1 (20%)
Time from diagnosis to Rituximab, months	34.0 (24.8–49.0)
Time from Rituximab to last follow-up, months	20.0 (10.3–23.4)

Data are presented as medians (interquartile ranges) or as numbers (percentages) unless otherwise indicated. Abbreviations: IIM, idiopathic inflammatory myopathies; ILD, interstitial lung disease; PM, polymyositis; DM, dermatomyositis; HRCT, high-resolution computed tomography; NSIP, nonspecific interstitial pneumonia; OP, organizing pneumonia.

**Table 2:** Treatment details, lung function, and inflammatory markers for IIM-ILD patients at time of rituximab.

Characteristics	Total
Case no.	8
Corticosteroid	8 (100%)
Prednisolone equivalent dose, mg	7.5 (5.0–7.5)
Duration, month	16.0 (15.0–47.5)
Cyclophosphamide	4 (50%)
Cumulative dose, mg	3275.0 (3088.0–3425.0)
Mycophenolate mofetil	1 (13%)
Dose, g	2.0
Duration, month	10.0
Methotrexate	1 (13%)
Dose, mg	7.5
Duration, month	40.0
Lung function	
FVC, %	48.5 (37.1–56.6)
FVC, L	1.6 (1.0–2.4)
DLco, %	34.0 (24.0–50.0)
DLco, mL/min/mmHg	5.2 (3.4–8.9)
Laboratory findings	
ESR, mm/h	116.0 (91.0–120.0)
CRP, mg/dL	0.58 (0.06–1.25)
WBC count, cells/μL	10,250 (5050–12,150)
LDH, U/L	342.5 (310.5–471.3)
CK, U/L	755.5 (72.5–2022.3)
Procalcitonin, ng/mL	0.03 (0.03–0.10)

Data are presented as medians (interquartile ranges) or as numbers (percentages) unless otherwise indicated. Abbreviations: IIM, idiopathic inflammatory myopathies; ILD, interstitial lung disease; FVC, forced vital capacity; DLco, diffusing capacity for carbon monoxide; ESR, erythrocyte sedimentation rate; CRP, C-reactive protein; WBC, white blood cell; LDH, lactate dehydrogenase; CK, creatine kinase.

**Table 3:** Serial lung function before and after rituximab in eight cases of IIM-ILD.

	FVC				DLco			
	% Predicted	*p*-Value *	L	*p*-Value *	% Predicted	*p*-Value *	mL/min /mmHg	*p*-Value *
Before 12 months	57.0 (39.7–75.2)	0.500	2.1 (1.2–3.1)	0.500	37.5 (21.0–58.5)	0.786	6.7 (3.2–10.2)	0.345
Before 6 months	54.1 (43.9–71.5)	0.043	1.8 (1.3–3.1)	0.046	39.0 (22.0–59.0)	0.398	7.3 (4.3–9.1)	0.352
Baseline	48.5 (36.4–64.1)		1.6 (1.0–2.7)		34.0 (22.3–40.3)		5.2 (3.2–8.2)	
After 6 months	54.9 (48.0–62.8)	0.128	1.9 (1.4–2.7)	0.091	37.5 (18.0–46.0)	0.600	6.7 (2.7–8.3)	0.753
After 12 months	54.1 (48.8–58.7)	0.116	1.8 (1.4–3.5)	0.116	37.5 (28.0–48.0)	0.343	7.8 (4.8–9.2)	0.138

Data are presented as medians with interquartile ranges. * p values were estimated using the Wilcoxon signed-rank test at baseline and at each time point. Abbreviations: IIM, idiopathic inflammatory myopathy; ILD, interstitial lung disease; FVC, forced vital capacity; DLco, diffusing capacity of the lung for carbon monoxide.

## Data Availability

All data generated and/or analyzed during the current study are available from the corresponding author upon reasonable request.

## Supplementary Materials

The following supporting information can be downloaded at: https://www.mdpi.com/article/10.3390/jcm12103406/s1, Table S1: Overview of treatment for each IIM-ILD patients. IIM, idiopathic inflammatory myopathies; ILD, interstitial lung disease; F, female; PM, polymyositis; DM, dermatomyositis; HTN, hypertension; AZA, azathioprine; GC, glucocorticoids; IVIG, intravenous immunoglobulin; MTX, methotrexate; CYC, cyclophosphamide; MMF, mycophenolate mofetil; CS, cyclosporine; Table S2: Serial lung function before and after rituximab in five IIM-ILD patients. Data are presented as medians with interquartile ranges. * *p* values were estimated using the Wilcoxon signed-rank test at baseline and at each time point. IIM, idiopathic inflammatory myopathy; ILD, interstitial lung disease; FVC, forced vital capacity; DLco, diffusing capacity of the lung for carbon monoxide; mo, month.
