# Supplementary material for: The Efficacy and Safety of Rituximab in Patients with Idiopathic Inflammatory Myopathy-Associated Interstitial Lung Disease: Case Series
Source: J Clin Med. 2023 May 11;12(10):3406. doi: 10.3390/jcm12103406 (PMC10219281; doi:10.3390/jcm12103406)
Supplement: Supplementary file 1 [file jcm-12-03406-s001.zip › JCM_Supplementary Table S1_Rituximab IIM.pdf]

Supplementary Table S1. Overview of treatment for each IIM-ILD patients.

Patient number	Age	Sex	Diagnosis	Co-morbidities	Total number of cycles	Total dose of rituximab (mg)	Time interval since last cycle (months)	Treatments prior to rituximab	Treatments between cycles	Duration of follow-up (months)
1	59	F	PM	Diabetes mellitus, Rectal cancer	2	2240	-	AZA, GC, IVIG, MTX	-	-
						1100	24	-	GC, IVIG	18
2	69	F	PM	Thyroid cancer	3	1100	-	CYC, GC, IVIG, MMF	-	-
						1600	10	-	GC	-
						2000	15	-	GC	8
3	38	F	DM	Diabetes mellitus	1	1900	-	AZA, CS, CYC, GC, IVIG, MTX, Tacrolimus	-	24
4	40	F	DM	-	1	1800	-	AZA, CYC, GC, IVIG, Tacrolimus	-	23
5	38	F	DM	HTN	1	1800	-	CS, CYC, GC	-	11

IIM, idiopathic inflammatory myopathies; ILD, interstitial lung disease; F, female; PM, polymyositis; DM, dermatomyositis; HTN, hypertension; AZA, azathioprine; GC, glucocorticoids; IVIG, intravenous immunoglobulin; MTX, methotrexate; CYC, cyclophosphamide; MMF, mycophenolate mofetil; CS, cyclosporine
