# Supplementary material for: The Efficacy and Safety of Rituximab in Patients with Idiopathic Inflammatory Myopathy-Associated Interstitial Lung Disease: Case Series
Source: J Clin Med. 2023 May 11;12(10):3406. doi: 10.3390/jcm12103406 (PMC10219281; doi:10.3390/jcm12103406)
Supplement: Supplementary file 1 [file jcm-12-03406-s001.zip › JCM_Supplementary Table S2_Rituximab IIM.pdf]

Supplementary Table S2: Serial lung function before and after rituximab in five IIM-ILD patients.

	FVC				DLco			
	% predicted	*P*-value*	L	*P*-value*	% predicted	*P*-value*	ml/min/mmHg	*P*-value*
Pre 12 mo	71.3 (41.4-71.3)	0.109	3.0 (1.2-3.0)	0.285	51.0 (22.0-51.0)	0.414	9.8 (3.8-9.85)	0.109
Pre 6 mo	53.5 (46.7-78.1)	0.080	1.9 (1.5-3.2)	0.080	39.0 (18.0-55.0)	0.893	9.0 (3.4-10.7)	0.786
Baseline	48.5 (39.6-71.6)		1.8 (1.3-2.9)		34.0 (24.0-53.0)		7.9 (4.3-9.9)	
Post 6 mo	55.1 (51.8-76.5)	0.043	2.0 (1.6-3.1)	0.043	39.0 (32.5-54.5)	0.138	8.3 (6.0-9.9)	0.225
Post 12 mo	54.6 (51.2-85.7)	0.043	2.0 (1.6-3.5)	0.043	40.0 (32.5-55.0)	0.066	8.9 (6.0-10.5)	0.068

Data are presented as medians with interquartile ranges. * *P value*s were estimated using the Wilcoxon signed-rank test at baseline and at each time point. Abbreviations: IIM, idiopathic inflammatory myopathy; ILD, interstitial lung disease; FVC, forced vital capacity; DLco, diffusing capacity of the lung for carbon monoxide; mo, month.
